# What is quality in long covid care? Lessons from a national quality improvement collaborative and multi-site ethnography

**DOI:** 10.1186/s12916-024-03371-6

**Published:** 2024-04-15

**Authors:** Trisha Greenhalgh, Julie L. Darbyshire, Cassie Lee, Emma Ladds, Jenny Ceolta-Smith

**Affiliations:** 1 Nuffield Department of Primary Care Health Sciences, University of Oxford, Woodstock Rd, Oxford, OX2 6GG UK; 2 Imperial College Healthcare NHS Trust, London, UK; 3 LOCOMOTION Patient Advisory Group and Lived Experience Representative, London, UK

**Keywords:** Long covid, Covid-19, Post-covid-19 syndrome, Quality improvement, Breakthrough collaboratives, Warranted variation, Unwarranted variation, Improvement science, Ethnography, Idiographic reasoning, Nomothetic reasoning

## Abstract

**Background:**

Long covid (post covid-19 condition) is a complex condition with diverse manifestations, uncertain prognosis and wide variation in current approaches to management. There have been calls for formal quality standards to reduce a so-called “postcode lottery” of care. The original aim of this study—to examine the nature of quality in long covid care and reduce unwarranted variation in services—evolved to focus on examining the reasons why standardizing care was so challenging in this condition.

**Methods:**

In 2021–2023, we ran a quality improvement collaborative across 10 UK sites. The dataset reported here was mostly but not entirely qualitative. It included data on the origins and current context of each clinic, interviews with staff and patients, and ethnographic observations at 13 clinics (50 consultations) and 45 multidisciplinary team (MDT) meetings (244 patient cases). Data collection and analysis were informed by relevant lenses from clinical care (e.g. evidence-based guidelines), improvement science (e.g. quality improvement cycles) and philosophy of knowledge.

**Results:**

Participating clinics made progress towards standardizing assessment and management in some topics; some variation remained but this could usually be explained. Clinics had different histories and path dependencies, occupied a different place in their healthcare ecosystem and served a varied caseload including a high proportion of patients with comorbidities. A key mechanism for achieving high-quality long covid care was when local MDTs deliberated on unusual, complex or challenging cases for which evidence-based guidelines provided no easy answers. In such cases, collective learning occurred through *idiographic (case-based) reasoning*, in which practitioners build lessons from the particular to the general. This contrasts with the *nomothetic reasoning* implicit in evidence-based guidelines, in which reasoning is assumed to go from the general (e.g. findings of clinical trials) to the particular (management of individual patients).

**Conclusion:**

Not all variation in long covid services is unwarranted. Largely because long covid’s manifestations are so varied and comorbidities common, generic “evidence-based” standards require much individual adaptation. In this complex condition, quality improvement resources may be productively spent supporting MDTs to optimise their case-based learning through interdisciplinary discussion. Quality assessment of a long covid service should include review of a sample of individual cases to assess how guidelines have been interpreted and personalized to meet patients’ unique needs.

**Study registration:**

NCT05057260, ISRCTN15022307.

## Background

### Long covid

The term “long covid” [1] means prolonged symptoms following SARS-CoV-2 infection not explained by an alternative diagnosis [2]. It embraces the US term “post-covid conditions” (symptoms beyond 4 weeks) [3], the UK terms “ongoing symptomatic covid-19” (symptoms lasting 4–12 weeks) and “post covid-19 syndrome” (symptoms beyond 12 weeks) [4] and the World Health Organization’s “post covid-19 condition” (symptoms occurring beyond 3 months and persisting for at least 2 months) [5]. Long covid thus defined is extremely common. In UK, for example, 1.8 million of a population of 67 million met the criteria for long covid in early 2023 and 41% of these had been unwell for more than 2 years [6].

Long covid is characterized by a constellation of symptoms which may include breathlessness, fatigue, muscle and joint pain, chest pain, memory loss and impaired concentration (“brain fog”), sleep disturbance, depression, anxiety, palpitations, dizziness, gastrointestinal problems such as diarrhea, skin rashes and allergy to food or drugs [2]. These lead to difficulties with essential daily activities such as washing and dressing, impaired exercise tolerance and ability to work, and reduced quality of life [2, 7, 8]. Symptoms typically cluster (e.g. in different patients, long covid may be dominated by fatigue, by breathlessness or by palpitations and dizziness) [9, 10]. Long covid may follow a fairly constant course or a relapsing and remitting one, perhaps with specific triggers [11]. Overlaps between fatigue-dominant subtypes of long covid, myalgic encephalomyelitis and chronic fatigue syndrome have been hypothesized [12] but at the time of writing remain unproven.

Long covid has been a contested condition from the outset. Whilst long-term sequelae following other coronavirus (SARS and MERS) infections were already well-documented [13], SARS-CoV-2 was originally thought to cause a short-lived respiratory illness from which the patient either died or recovered [14]. Some clinicians dismissed protracted or relapsing symptoms as due to anxiety or deconditioning, especially if the patient had not had laboratory-confirmed covid-19. People with long covid got together in online groups and shared accounts of their symptoms and experiences of such “gaslighting” in their healthcare encounters [15, 16]. Some groups conducted surveys on their members, documenting the wide range of symptoms listed in the previous paragraph and showing that whilst long covid is more commonly a sequel to severe acute covid-19, it can (rarely) follow a mild or even asymptomatic acute infection [17].

Early publications on long covid depicted a post-pneumonia syndrome which primarily affected patients who had been hospitalized (and sometimes ventilated) [18, 19]. Later, covid-19 was recognized to be a multi-organ inflammatory condition (the pneumonia, for example, was reclassified as *pneumonitis*) and its long-term sequelae attributed to a combination of viral persistence, dysregulated immune response (including auto-immunity), endothelial dysfunction and immuno-thrombosis, leading to damage to the lining of small blood vessels and (thence) interference with transfer of oxygen and nutrients to vital organs [20–24]. But most such studies were highly specialized, laboratory-based and written primarily for an audience of fellow laboratory researchers. Despite demonstrating mean differences in a number of metabolic variables, they failed to identify a reliable biomarker that could be used routinely in the clinic to rule a diagnosis of long covid in or out. Whilst the evidence base from laboratory studies grew rapidly, it had little influence on clinical management—partly because most long covid clinics had been set up with impressive speed by front-line clinical teams to address an immediate crisis, with little or no input from immunologists, virologists or metabolic specialists [25].

Studies of the patient experience revealed wide geographical variation in whether any long covid services were provided and (if they were) which patients were eligible for these and what tests and treatments were available [26]. An interim UK clinical guideline for long covid had been produced at speed and published in December 2020 [27], but it was uncertain about diagnostic criteria, investigations, treatments and prognosis. Early policy recommendations for long covid services in England, based on wide consultation across UK, had proposed a tiered service with “tier 1” being supported self-management, “tier 2” generalist assessment and management in primary care, “tier 3” specialist rehabilitation or respiratory follow-up with oversight from a consultant physician and “tier 4” tertiary care for patients with complications or complex needs [28]. In 2021, ring-fenced funding was allocated to establish 90 multidisciplinary long covid clinics in England [29]; some clinics were also set up with local funding in Scotland and Wales. These clinics varied widely in eligibility criteria, referral pathways, staffing mix (some had no doctors at all) and investigations and treatments offered. A further policy document on improving long covid services was published in 2022 [30]; it recommended that specialist long covid clinics should continue, though the long-term funding of these services remains uncertain [31]. To build the evidence base for delivering long covid services, major programs of publicly funded research were commenced in both UK [32] and USA [33].

In short, at the time this study began (late 2021), there appeared to be much scope for a program of quality improvement which would capture fast-emerging research findings, establish evidence-based standards and ensure these were rapidly disseminated and consistently adopted across both specialist long covid services and in primary care.

### Quality improvement collaboratives

The quality improvement movement in healthcare was born in the early 1980s when clinicians and policymakers US and UK [34–37] began to draw on insights from outside the sector [38–40]. Adapting a total quality management approach that had previously transformed the Japanese car industry, they sought to improve efficiency, reduce waste, shift to treating the upstream causes of problems (hence preventing disease) and help all services approach the standards of excellence achieved by the best. They developed an approach based on (a) understanding healthcare as a complex system (especially its key interdependencies and workflows), (b) analysing and addressing variation within the system, (c) learning continuously from real-world data and (d) developing leaders who could motivate people and help them change structures and processes [41–44].

Quality improvement collaboratives (originally termed “breakthrough collaboratives” [45]), in which representatives from different healthcare organizations come together to address a common problem, identify best practice, set goals, share data and initiate and evaluate improvement efforts [46], are one model used to deliver system-wide quality improvement. It is widely assumed that these collaboratives work because—and to the extent that—they identify, interpret and implement high-quality evidence (e.g. from randomized controlled trials).

Research on why quality improvement collaboratives succeed or fail has produced the following list of critical success factors: taking a whole-system approach, selecting a topic and goal that fits with organizations’ priorities, fostering a culture of quality improvement (e.g. that quality is everyone’s job), engagement of everyone (including the multidisciplinary clinical team, managers, patients and families) in the improvement effort, clearly defining people’s roles and contribution, engaging people in preliminary groundwork, providing organizational-level support (e.g. chief executive endorsement, protected staff time, training and support for teams, resources, quality-focused human resource practices, external facilitation if needed), training in specific quality improvement techniques (e.g. plan-do-study-act cycle), attending to the human dimension (including cultivating trust and working to ensure shared vision and buy-in), continuously generating reliable data on both processes (e.g. current practice) and outcomes (clinical, satisfaction) and a “learning system” infrastructure in which knowledge that is generated feeds into individual, team and organizational learning [47–54].

The quality improvement collaborative approach has delivered many successes but it has been criticized at a theoretical level for over-simplifying the social science of human motivation and behaviour and for adopting a somewhat mechanical approach to the study of complex systems [55, 56]. Adaptations of the original quality improvement methodology (e.g. from Sweden [57, 58]) have placed greater emphasis on human values and meaning-making, on the grounds that reducing the complexities of a system-wide quality improvement effort to a set of abstract and generic “success factors” will miss unique aspects of the case such as historical path dependencies, personalities, framing and meaning-making and micropolitics [59].

Perhaps this explains why, when the abovementioned factors are met, a quality improvement collaborative’s success is more likely but is not guaranteed, as a systematic review demonstrated [60]. Some well-designed and well-resourced collaboratives addressing clear knowledge gaps produced few or no sustained changes in key outcome measures [49, 53, 60–62]. To identify why this might be, a detailed understanding of a service’s history, current challenges and contextual constraints is needed. This explains our decision, part-way through the study reported here, to collect rich contextual data on participating sites so as to better explain success or failure of our own collaborative.

### Warranted and unwarranted variation in clinical practice

A generation ago, Wennberg described most variation in clinical practice as “unwarranted” (which he defined as variation in the utilization of health care services that cannot be explained by variation in patient illness or patient preferences) [63]. Others coined the term “postcode lottery” to depict how such variation allegedly impacted on health outcomes [64]. Wennberg and colleagues’ *Atlas of Variation*, introduced in 1999 [65], and its UK equivalent, introduced in 2010 [66], described wide regional differences in the rates of procedures from arthroscopy to hysterectomy, and were used to prompt services to identify and address examples of under-treatment, mis-treatment and over-treatment. Numerous similar initiatives, mostly based on hospital activity statistics, have been introduced around the world [66–69]. Sutherland and Levesque’s proposed framework for analysing variation, for example, has three domains: *capacity* (broadly, whether sufficient resources are allocated at organizational level and whether individuals have the time and headspace to get involved), *evidence* (the extent to which evidence-based guidelines exist and are followed), and *agency* (e.g. whether clinicians are engaged with the issue and the effect of patient choice) [70].

Whilst it is clearly a good idea to identify unwarranted variation in practice, it is also important to acknowledge that variation can be *warranted*. The very act of measuring and describing variation carries great rhetorical power, since revealing geographical variation in any chosen metric effectively frames this as a problem with a conceptually simple solution (reducing variation) that will appeal to both politicians and the public [71]. The temptation to expose variation (e.g. via visualizations such as maps) and address it in mechanistic ways should be resisted until we have fully understood the reasons why it exists, which may include perverse incentives, insufficient opportunities to discuss cases with colleagues, weak or absent feedback on practice, unclear decision processes, contested definitions of appropriate care and professional challenges to guidelines [72].

## Research question, aims and objectives

### Research question

What is quality in long covid care and how can it best be achieved?

### Aims

1. To identify best practice and reduce unwarranted variation in UK long covid services.
2. To explain aspects of variation in long covid services that are or may be warranted.

### Objectives

Our original objectives were to:

1. Establish a quality improvement collaborative for 10 long covid clinics across UK.
2. Use quality improvement methods in collaboration with patients and clinic staff to prioritize aspects of care to improve. For each priority topic, identify best (evidence-informed) clinical practice, measure performance in each clinic, compare performance with a best practice benchmark and improve performance.
3. Produce organizational case studies of participating long covid clinics to explain their origins, evolution, leadership, ethos, population served, patient pathways and place in the wider healthcare ecosystem.
4. Examine these case studies to explain variation in practice, especially in topics where the quality improvement cycle proves difficult to follow or has limited impact.

## Methods

### The LOCOMOTION study

LOCOMOTION (LOng COvid Multidisciplinary consortium Optimising Treatments and services across the NHS) was a 30-month multi-site case study of 10 long covid clinics (8 in England, 1 in Wales and 1 in Scotland), beginning in 2021, which sought to optimise long covid care. Each clinic offered multidisciplinary care to patients referred from primary or secondary care (and, in some cases, self-referred), and held regular multidisciplinary team (MDT) meetings, mostly online via Microsoft Teams, to discuss cases. A study protocol for LOCOMOTION, with details of ethical approvals, management, governance and patient involvement has been published [25]. The three main work packages addressed quality improvement, technology-supported patient self-management and phenotyping and symptom clustering. This paper reports on the first work package, focusing mainly on qualitative findings.

### Setting up the quality improvement collaborative

We broadly followed standard methodology for “breakthrough” quality improvement collaboratives [44, 45], with two exceptions. First, because of geographical distance, continuing pandemic precautions and developments in videoconferencing technology, meetings were held online. Second, unlike in the original breakthrough model, patients were included in the collaborative, reflecting the cultural change towards patient partnerships since the model was originally proposed 40 years ago.

Each site appointed a clinical research fellow (doctor, nurse or allied health professional) funded partly by the LOCOMOTION study and partly with clinical sessions; some were existing staff who were backfilled to take on a research role whilst others were new appointments. The quality improvement meetings were held approximately every 8 weeks on Microsoft Teams and lasted about 2 h; there was an agenda and a chair, and meetings were recorded with consent. The clinical research fellow from each clinic attended, sometimes joined by the clinical lead for that site. In the initial meeting, the group proposed and prioritized topics before merging their consensus with the list of priority topics generated separately by patients (there was much overlap but also some differences).

In subsequent meetings, participants attempted to reach consensus on how to define, measure and achieve quality for each priority topic in turn, implement this approach in their own clinic and monitor its impact. Clinical leads prepared illustrative clinical cases and summaries of the research evidence, which they presented using Microsoft Powerpoint; the group then worked towards consensus on the implications for practice through general discussion. Clinical research fellows assisted with literature searches, collected baseline data from their own clinic, prepared and presented anonymized case examples, and contributed to collaborative goal-setting for improvement. Progress on each topic was reviewed at a later meeting after an agreed interval.

An additional element of this work package was semi-structured interviews with 29 patients, recruited from 9 of the 10 participating sites, about their clinic experiences with a view to feeding into service improvement (in the other site, no patient volunteered).

Our patient advisory group initially met separately from the quality improvement collaborative. They designed a short survey of current practice and sent it to each clinic; the results of this informed a prioritization exercise for topics where they considered change was needed. The patient-generated list was tabled at the quality improvement collaborative discussions, but patients were understandably keen to join these discussions directly. After about 9 months, some patient advisory group members joined the regular collaborative meetings. This dynamic was not without its tensions, since sharing performance data requires trust and there were some concerns about confidentiality when real patient cases were discussed with other patients present.

### How evidence-informed quality targets were set

At the time the study began, there were no published large-scale randomized controlled trials of any interventions for long covid. We therefore followed a model used successfully in other quality improvement efforts where research evidence was limited or absent or it did not translate unambiguously into models for current services. In such circumstances, the best evidence may be custom and practice in the best-performing units. The quality improvement effort becomes oriented to what one group of researchers called “potentially better practices”—that is, practices that are “developed through analysis of the processes of care, literature review, and site visits” (page 14) [73]. The idea was that facilitated discussion among clinical teams, drawing on published research where available but also incorporating clinical experience, established practice and systematic analysis of performance data across participating clinics would surface these “potentially better practices”—an approach which, though not formally tested in controlled trials, appears to be associated with improved outcomes [46, 73].

### Adding an ethnographic component

Following limited progress made on some topics that had been designated high priority, we interviewed all 10 clinical research fellows (either individually or, in two cases, with a senior clinician present) and 18 other clinic staff (five individually plus two groups of 5 and 8), along with additional informal discussions, to explore the challenges of implementing the changes that had been agreed. These interviews were not audiotaped but detailed notes were made and typed up immediately afterwards. It became evident that some aspects of what the collaborative had deemed “evidence-informed” care were contested by front-line clinic staff, perceived as irrelevant to the service they were delivering, or considered impossible to implement. To unpack these issues further, the research protocol was amended to include an ethnographic component.

TG and EL (academic general practitioners) and JLD (a qualitative researcher with a PhD in the patient experience) attended a total of 45 MDT meetings in participating clinics (mostly online or hybrid). Staff were informed in advance that there would be an observer present; nobody objected. We noted brief demographic and clinical details of cases discussed (but no identifying data), dilemmas and uncertainties on which discussions focused, and how different staff members contributed.

TG made 13 in-person visits to participating long covid clinics. Staff were notified in advance; all were happy to be observed. Visits lasted between 5 and 8 h (54 h in total). We observed support staff booking patients in and processing requests and referrals, and shadowed different clinical staff in turn as they saw patients. Patients were informed of our presence and its purpose beforehand and given the opportunity to decline (three of 53 patients approached did). We discussed aspects of each case with the clinician after the patient left. When invited, we took breaks with staff and used these as an opportunity to ask them informally what it was like working in the clinic.

Ethnographic observation, analysis and reporting was geared to generating a rich interpretive account of the clinical, operational and interpersonal features of each clinic—what Van Maanen calls an “impressionist tales” [74]. Our work was also guided by the principles set out by Golden-Biddle and Locke, namely authenticity (spending time in the field and basing interpretations on these direct observations), plausibility (creating a plausible account through rich persuasive description) and criticality (e.g. reflexively examining our own assumptions) [75]. Our collection and analysis of qualitative data was informed by our own professional backgrounds (two general practitioners, one physical therapist, two non-clinicians).

In both MDTs and clinics, we took contemporaneous notes by hand and typed these up immediately afterwards.

### Data management and analysis

Typed interview notes and field notes from clinics were collated in a set of Word documents, one for each clinic attended. They were analysed thematically [76] with attention to the literature on quality improvement and variation (see “Background”). Interim summaries were prepared on each clinic, setting out the narrative of how it had been established, its ethos and leadership, setting and staffing, population served and key links with other parts of the local healthcare ecosystem.

Minutes and field notes from the quality improvement collaborative meetings were summarized topic by topic, including initial data collected by the researchers-in-residence, improvement actions taken (or attempted) in that clinic, and any follow-up data shared. Progress or lack of it was interpreted in relation to the contextual case summary for that clinic.

Patient cases seen in clinic, and those discussed by MDTs, were summarized as brief case narratives in Word documents. Using the constant comparative method [77], we produced an initial synthesis of the clinical picture and principles of management based on the first 10 patient cases seen, and refined this as each additional case was added. Demographic and brief clinical and social details were also logged on Excel spreadsheets. When writing up clinical cases, we used the technique of composite case construction (in which we drew on several actual cases to generate a fictitious one, thereby protecting anonymity whilst preserving key empirical findings [78]); any names reported in this paper are pseudonyms.

### Member checking

A summary was prepared for each clinic, including a narrative of the clinic’s own history and a summary of key quality issues raised across the ten clinics. These summaries included examples from real cases in our dataset. These were shared with the clinical research fellow and a senior clinician from the clinic, and amended in response to feedback. We also shared these summaries with representatives from the patient advisory group.

## Results

### Overview of dataset

This study generated three complementary datasets. First, the video recordings, minutes, and field notes of 12 quality improvement collaborative meetings, along with the evidence summaries prepared for these meetings and clinic summaries (e.g. descriptions of current practice, audits) submitted by the clinical research fellows. This dataset illustrated wide variation in practice, and (in many topics) gaps or ambiguities in the evidence base.

Second, interviews with staff (*n* = 30) and patients (*n* = 29) from the clinics, along with ethnographic field notes (approximately 100 pages) from 13 in-person clinic visits (54 h), including notes on 50 patient consultations (40 face-to-face, 6 telephone, 4 video). This dataset illustrated the heterogeneity among the ten participating clinics.

Third, field notes (approximately 100 pages), including discussions on 244 clinical cases from the 45 MDT meetings (49 h) that we observed. This dataset revealed further similarities and contrasts among clinics in how patients were managed. In particular, it illustrated how, for the complex patients whose cases were presented at these meetings, teams made sense of, and planned for, each case through multidisciplinary dialogue. This dialogue typically began with one staff member presenting a detailed clinical history along with a narrative of how it had affected the patient’s life and what was at stake for them (e.g. job loss), after which professionals from various backgrounds (nursing, physical therapy, occupational therapy, psychology, dietetics, and different medical specialties) joined in a discussion about what to do.

The ten participating sites are summarized in Table 1.

**Table 1:** participating sites (Sites C, E and F are described in more detail in the text)

Site and jurisdiction	Brief description	Typical patient pathway and experience	MDT meetings observed (hours, patients)	Clinics observed (hours, patients)	Total patients and age range
A (England)	GP-led clinic with a core MDT including nurse, physio, OT, psych	Patients referred by GPs have an initial telephone assessment, usually nurse-led. Follow up appointments are virtual by default, although home visits can be arranged. Some patients are selected for review at the MDT, where a suggested plan for investigation/ management/ onward referral) is made and forwarded to the patient’s GP	7 MDTs (7 h, 17 patients)	2 clinics (5 h); 3 patients	20 patients aged 30–62
B (Wales)	Hospital-based clinic run by respiratory consultant and clinical research fellow	Accepts referrals from primary and secondary care. All referrals triaged by consultant. No formal MDT support but input available on request from community-based rehabilitation team. Most initial consults are face-to-face with follow ups virtual or face-to-face, based on need	3 MDTs (3 h, 22 patients)	Not visited	22 patients aged 30–62
C (England)	Community clinic, physically based in community health offices but run virtually. Led jointly by GP and OT, with physio, psych and MH. Wider MDT includes SALT and dietician	Patients referred by GPs have a 60–90-min telephone or video consultation with GP or OT. GP (with MDT input) makes decisions about further investigation or onward referral but there is no facility for patients to be assessed in-person by the long covid team	5 MDTs (7 h, 37 patients)	1 clinic (6 h); 5 patients	42 patients aged 21–81
D (Scotland)	Hospital clinic led by clinical psychologist with support from infectious disease consultant. MDT also includes GP, physio, OT	Because clinic serves a wide geographical area, most patients have a video or telephone consultation; there is a small capacity for in-person clinic reviews. Much ongoing support is virtual	5 MDTs (8 h, 54 patients)	Not visited	54 patients aged 18–70
E (England)	Hospital clinic based in chest and allergy outpatients. Set up by a respiratory consultant with interest in long covid but now multiple consultants rotate. With OT, physio, psych	Primarily an assessment service. Patients referred by GP are seen in person by 4 MDT members in turn; each case is discussed briefly before and after clinic. Good integration and pathways with community rehab services. Local services include specialist POTS clinic based in cardiology	2 MDTs (2 h, 15 patients)	1 clinic (7 h); 5 patients	20 patients aged 25–73
F (England)	Large rehabilitation service linked to a teaching hospital and university. Community (OT-led) clinic with physio, OT, psych, therapy assistants, dieticians. Close links to consultant-led clinic based in hospital rehabilitation unit	Community tier takes GP referrals and links with community-based rehab in a local leisure centre. Well-established referral links and pathways for cardiology, respiratory, neurology, and sleep services as well as local mental health and well-being services	3 MDTs (3 h, 17 patients)	2 clinics (7 h); 7 patients	24 patients aged 18–68
G (England)	Large hospital service based in respiratory outpatients in teaching hospital. Consultant-led with nurse practitioner support, plus large MDT including OT, physio, psych	Patients referred by GP have 60-min telephone assessment by nurse practitioner before seeing doctor in-person. Doctor orders further tests and refers on to other specialties and programs	9 MDTs (6 h, 30 patients)	2 clinics (8 h); 10 patients	40 patients aged 34–87
H (England)	Consultant-led clinic in large teaching hospital and university, originally established by respiratory consultant. Also, separate specialist POTS clinic run by cardiologist with special interest	At the time of the study, patients had a one-stop holistic assessment with input from medics, psych, physio and specialist lung function tests. Direct links into secondary care referral systems and a specialist rehab team. In September 2023 the clinic was remodeled; it now runs under infectious diseases with a focus on CFS/ME, with support from a specialist nurse	2 tier 4 MDTs (2 h, 13 patients)	2 clinics (8 h); 7 patients	20 patients aged 18–69
I (England)	Based in rehabilitation outpatients on a large hospital campus with strong research links. Led jointly by rehabilitation and respiratory consultants, with MDT including OT, physio, SEM, psych, MH	Patients referred by GP are seen by consultant for 40-min face-to-face assessment before seeing allied professionals who refer on to rehab programs. Allied health professionals include specialist pulmonary rehab physios, OTs with interest in ME/CFS, and community psychological support services	2 MDTs (3 h, 19 patients)	2 clinics (8 h); 9 patients	28 patients aged 26–72
J (England)	Multi-tier system across a large urbanized geographical area. Hospital tier is led by respiratory consultant with physio, OT, psych, MH, and rehabilitation medicine. Region-wide (tier 4) MDT includes endocrinology, cardiology, neurology, psychiatry and occupational health	Patients referred by GPs are seen in GP-led clinic, which offers in-person holistic assessment, management and rehabilitation. Selected cases are taken to the secondary care MDT (for consultant opinion) or the region-wide MDT (complex cases discussed by multiple specialists)	4 tier 3 MDTs (3 h, 11 patients) plus 3 tier 4 MDTs (5 h, 9 patients)	1 clinic (5 h); 4 patients	24 patients aged 25–71
TOTAL			45 MDTs (49 h, 244 patients)	13 clinics (54 h, 50 patients)	45 MDTs, 13 clinics (104 h, 294 patients)

*MDT *multidisciplnary team, *ME/CFS* Myalgic encephalomyelitis/chronic fatigue syndrome

In the next two sections, we explore two issues—difficulty defining best practice and the heterogeneous nature of the clinics—that were key to explaining why quality, when pursued in a 10-site collaborative, proved elusive. We then briefly summarize patients’ accounts of their experience in the clinics and give three illustrative examples of the elusiveness of quality improvement using selected topics that were prioritized in our collaborative: outcome measures, investigation of palpitations and management of fatigue. In the final section of the results, we describe how MDT deliberations proved crucial for *local* quality improvement. Further detail on clinical priority topics will be presented in a separate paper.

### “Best practice” in long covid: uncertainty and conflict

The study period (September 2021 to December 2023) corresponded with an exponential increase in published research on long covid. Despite this, the quality improvement collaborative found few unambiguous recommendations for practice. This gap between what the research literature offered and what clinical practice needed was partly ontological (relating what long covid *is*). One major bone of contention between patients and clinicians (also evident in discussions with our patient advisory group), for example, was how far (and in whom) clinicians should look for and attempt to treat the various metabolic abnormalities that had been documented in laboratory research studies. The literature on this topic was extensive but conflicting [20–24, 79–82]; it was heavy on biological detail but light on clinical application.

Patients were often aware of particular studies that appeared to offer plausible molecular or cellular explanations for symptom clusters along with a drug (often repurposed and off-label) whose mechanism of action appeared to be a good fit with the metabolic chain of causation. In one clinic, for example, we were shown an email exchange between a patient (not medically qualified) and a consultant, in which the patient asked them to reconsider their decision not to prescribe low-dose naltrexone, an opioid receptor antagonist with anti-inflammatory properties. The request included a copy of a peer-reviewed academic paper describing a small, uncontrolled pre-post study (i.e. a weak study design) in which this drug appeared to improve symptoms and functional performance in patients with long covid, as well as a mechanistic argument explaining why the patient felt this drug was a plausible choice in their own case.

This patient’s clinician, in common with most clinicians delivering front-line long covid services, considered that the evidence for such mechanism-based therapies was weak. Clinicians generally felt that this evidence, whilst promising, did not yet support routine measurement of clotting factors, antibodies, immune cells or other biomarkers or the prescription of mechanism-based therapies such as antivirals, anti-inflammatories or anticoagulants. Low-dose naltroxone, for example, is currently being tested in at least one randomized controlled trial (see National Clinical Trials Registry NCT05430152), which had not reported at the time of our observations.

Another challenge to defining best practice was the oft-repeated phrase that long covid is a “diagnosis by exclusion”, but the high prevalence of comorbidities meant that the “pure” long covid patient untainted by other potential explanations for their symptoms was a textbook ideal. In one MDT, for example, we observed a discussion about a patient who had had both swab-positive covid-19 *and* erythema migrans (a sign of Lyme disease) in the weeks before developing fatigue, yet local diagnostic criteria for each condition required the other to be excluded.

The logic of management in most participating clinics was pragmatic: prompt multidisciplinary assessment and treatment with an emphasis on obtaining a detailed clinical history (including premorbid health status), excluding serious complications (“red flags”), managing specific symptom clusters (for example, physical therapy for breathing pattern disorder), treating comorbidities (for example, anaemia, diabetes or menopause) and supporting whole-person rehabilitation [7, 83]. The evidentiary questions raised in MDT discussions (which did not include patients) addressed the practicalities of the rehabilitation model (for example, whether cognitive therapy for neurocognitive complications is as effective when delivered online as it is when delivered in-person) rather than the molecular or cellular mechanisms of disease. For example, the question of whether patients with neurocognitive impairment should be tested for micro-clots or treated with anticoagulants never came up in the MDTs we observed, though we did visit a tertiary referral clinic (the tier 4 clinic in site H), whose lead clinician had a research interest in inflammatory coagulopathies and offered such tests to selected patients.

Because long covid typically produces dozens of symptoms that tend to be uniquely patterned in each patient, the uncertainties on which MDT discussions turned were rarely about *general* evidence of the kind that might be found in a guideline (e.g. how should fatigue be managed?). Rather they concerned *particular* case-based clinical decisions (e.g. how should *this* patient’s fatigue be managed, given the specifics of this case?). An example from our field notes illustrates this:

*Physical therapist presents the case of a 39-year-old woman who works as a cleaner on an overnight ferry. Has had long covid for 2 years. Main symptoms are shortness of breath and possible anxiety attacks, especially when at work. She has had a course of physical therapy to teach diaphragmatic breathing but has found that focusing on her breathing makes her more anxious. Patient has to do a lot of bending in her job (e.g. cleaning toilets and under seats), which makes her dizzy, but Active Stand Test was normal. She also has very mild tricuspid incompetence [someone reads out a cardiology report—not hemodynamically significant].*

*Rehabilitation guidelines (e.g. WHO) recommend phased return to work (e.g. with reduced hours) and frequent breaks. “Tricky!” says someone. The job is intense and busy, and the patient can’t afford not to work. Discussion on whether all her symptoms can be attributed to tension and anxiety. Physical therapist who runs the breathing group says, “No, it’s long covid”, and describes severe initial covid-19 episode and results of serial chest X-rays which showed gradual clearing of ground glass shadows. Team discussion centers on how to negotiate reduced working hours in this particular job, given the overnight ferry shifts.*

*--MDT discussion, Site D*

This example raises important considerations about the nature of clinical knowledge in long covid. We return to it in the final section of the “Results” and in the “Discussion”.

### Long covid clinics: a heterogeneous context for quality improvement

Most participating clinics had been established in mid-2020 to follow up patients who had been hospitalized (and perhaps ventilated) for severe acute covid-19. As mass vaccination reduced the severity of acute covid-19 for most people, the patient population in all clinics progressively shifted to include fewer “post-ICU [intensive care unit]” patients (in whom respiratory symptoms almost always dominated), and more people referred by their general practitioners or other secondary care specialties who had not been hospitalized for their acute covid-19 infection, and in whom fatigue, brain fog and palpitations were often the most troubling symptoms. Despite these similarities, the ten clinics had very different histories, geographical and material settings, staffing structures, patient pathways and case mix, as Table 1 illustrates. Below, we give more detail on three example sites.

Site C was established as a generalist “assessment-only” service by a general practitioner with an interest in infectious diseases. It is led jointly by that general practitioner and an occupational therapist, assisted by a wide range of other professionals including speech and language therapy, dietetics, clinical psychology and community-based physical therapy and occupational therapy. It has close links with a chronic fatigue service and a pain clinic that have been running in the locality for over 20 years. The clinic, which is entirely virtual (staff consult either from home or from a small side office in the community trust building), is physically located in a low-rise building on the industrial outskirts of a large town, sharing office space with various community-based health and social care services. Following a 1-h telephone consultation by one of the clinical leads, each patient is discussed at the MDT and then either discharged back to their general practitioner with a detailed management plan or referred on to one of the specialist services. This arrangement evolved to address a particular problem in this locality—that many patients with long covid were being referred by their general practitioner to multiple specialties (e.g. respiratory, neurology, fatigue), leading to a fragmented patient experience, unnecessary specialist assessments and wasteful duplication. The generalist assessment by telephone is oriented to documenting what is often a complex illness narrative (including pre-existing physical and mental comorbidities) and working with the patient to prioritize which symptoms or problems to pursue in which order.

Site E, in a well-regarded inner-city teaching hospital, had been set up in 2020 by a respiratory physician. Its initial ethos and rationale had been “respiratory follow-up”, with strong emphasis on monitoring lung damage via repeated imaging and lung function tests and in ensuring that patients received specialist physical therapy to “re-learn” efficient breathing techniques. Over time, this site has tried to accommodate a more multi-system assessment, with the introduction of a consultant-led infectious disease clinic for patients without a dominant respiratory component, reflecting the shift towards a more fatigue-predominant case mix. At the time of our fieldwork, each patient was seen in turn by a physician, psychologist, occupational therapist and respiratory physical therapist (half an hour each) before all four staff reconvened in a face-to-face MDT meeting to form a plan for each patient. But whilst a wide range of patients with diverse symptoms were discussed at these meetings, there remained a strong focus on respiratory pathology (e.g. tracking improvements in lung function and ensuring that coexisting asthma was optimally controlled).

Site F, one of the first long covid clinics in UK, was set up by a rehabilitation consultant who had been drafted to work on the ICU during the first wave of covid-19 in early 2020. He had a longstanding research interest in whole-patient rehabilitation, especially the assessment and management of chronic fatigue and pain. From the outset, clinic F was more oriented to rehabilitation, including vocational rehabilitation to help patients return to work. There was less emphasis on monitoring lung function or pursuing respiratory comorbidities. At the time of our fieldwork, clinic F offered both a community-based service (“tier 2”) led by an occupational therapist, supported by a respiratory physical therapist and psychologist, and a hospital-based service (“tier 3”) led by the rehabilitation consultant, supported by a wider MDT. Staff in both tiers emphasized that each patient needs a full physical and mental assessment and help to set and work towards achievable goals, whilst staying within safe limits so as to avoid post-exertional symptom exacerbation. Because of the research interest of the lead physician, clinic F adapted well to the growing numbers of patients with fatigue and quickly set up research studies on this cohort [84].

Details of the other seven sites are shown in Table 1. Broadly speaking, sites B, E, G and H aligned with the “respiratory follow-up” model and sites F and I aligned with the “rehabilitation” model. Sites A and J had a high-volume, multi-tiered service whose community tier aligned with the “holistic GP assessment” model (site C above) and which also offered a hospital-based, rehabilitation-focused tier. The small service in Scotland (site D) had evolved from an initial respiratory focus to become part of the infectious diseases (ME/CFS) service; Lyme disease (another infectious disease whose sequelae include chronic fatigue) was also prevalent in this region.

### The patient experience

Whilst the 10 participating clinics were very diverse in staffing, ethos and patient flows, the 29 patient interviews described remarkably consistent clinic experiences. Almost all identified the biggest problem to be the extended wait of several months before they were seen and the limited awareness (when initially referred) of what long covid clinics could provide. Some talked of how they cried with relief when they finally received an appointment. When the quality improvement collaborative was initially established, waiting times and bottlenecks were patients’ the top priority for quality improvement, and this ranking was shared by clinic staff, who were very aware of how much delays and uncertainties in assessment and treatment compounded patients’ suffering. This issue resolved to a large extent over the study period in all clinics as the referral backlog cleared and the incidence of new cases of long covid fell [85]; it will be covered in more detail in a separate publication.

Most patients in our sample were satisfied with the care they received when they were finally seen in clinic, especially how they finally felt “heard” after a clinician took a full history. They were relieved to receive affirmation of their experience, a diagnosis of what was wrong and reassurance that they were believed. They were grateful for the input of different members of the multidisciplinary teams and commented on the attentiveness, compassion and skill of allied professionals in particular (“she was wonderful, she got me breathing again”—patient BIR145 talking about a physical therapist). One or two patient participants expressed confusion about who exactly they had seen and what advice they had been given, and some did not realize that a telephone assessment had been an actual clinical consultation. A minority expressed disappointment that an expected investigation had not been ordered (one commented that they had not had any blood tests at all). Several had assumed that the help and advice from the long covid clinic would continue to be offered until they were better and were disappointed that they had been discharged after completing the various courses on offer (since their clinic had been set up as an “assessment only” service).

In the next sections, we give examples of topics raised in the quality improvement collaborative and how they were addressed.

### Example quality topic 1: Outcome measures

The first topic considered by the quality improvement collaborative was how (that is, using which measures and metrics) to assess and monitor patients with long covid. In the absence of a validated biomarker, various symptom scores and quality of life scales—both generic and disease-specific—were mooted. Site F had already developed and validated a patient-reported outcome measure (PROM), the C19-YRS (Covid-19 Yorkshire Rehabilitation Scale) and used it for both research and clinical purposes [86]. It was quickly agreed that, for the purposes of generating comparative research findings across the ten clinics, the C19-YRS should be used at all sites and completed by patients three-monthly. A commercial partner produced an electronic version of this instrument and an app for patient smartphones. The quality improvement collaborative also agreed that patients should be asked to complete the EUROQOL EQ5D, a widely used generic health-related quality of life scale [87], in order to facilitate comparisons between long covid and other chronic conditions.

In retrospect, the discussions which led to the unopposed adoption of these two measures as a “quality” initiative in clinical care were somewhat aspirational. A review of progress at a subsequent quality improvement meeting revealed considerable variation among clinics, with a wide variety of measures used in different clinics to different degrees. Reasons for this variation were multiple. First, although our patient advisory group were keen that we should gather as much data as possible on the patient experience of this new condition, many clinic patients found the long questionnaires exhausting to complete due to cognitive impairment and fatigue. In addition, whilst patients were keen to answer questions on symptoms that troubled them, many had limited patience to fill out repeated surveys on symptoms that did not trouble them (“it almost felt as if I’ve not got long covid because I didn’t feel like I fit the criteria as they were laying it out”—patient SAL001). Staff assisted patients in completing the measures when needed, but this was time-consuming (up to 45 min per instrument) and burdensome for both staff and patients. In clinics where a high proportion of patients required assistance, staff time was the rate-limiting factor for how many instruments got completed. For some patients, one short instrument was the most that could be asked of them, and the clinician made a judgement on which one would be in their best interests on the day.

The second reason for variation was that the clinical diagnosis and management of particular features, complications and comorbidities of long covid required more nuance than was provided by these relatively generic instruments, and the level of detail sought varied with the specialist interest of the clinic (and the clinician). The modified C19-YRS [88], for example, contained 19 items, of which one asked about sleep quality. But if a patient had sleep difficulties, many clinicians felt that these needed to be documented in more detail—for example using the 8-item Epworth Sleepiness Scale, originally developed for conditions such as narcolepsy and obstructive sleep apnea [89]. The “Epworth score” was essential currency for referrals to some but not all specialist sleep services. Similarly, the C19-YRS had three items relating to anxiety, depression and post-traumatic stress disorder, but in clinics where there was a strong focus on mental health (e.g. when there was a resident psychologist), patients were usually invited to complete more specific tools (e.g. the Patient Health Questionnaire 9 [90], a 9-item questionnaire originally designed to assess severity of depression).

The third reason for variation was custom and practice. Ethnographic visits revealed that paper copies of certain instruments were routinely stacked on clinicians’ desks in outpatient departments and also (in some cases) handed out by administrative staff in waiting areas so that patients could complete them before seeing the clinician. These familiar clinic artefacts tended to be short (one-page) instruments that had a long tradition of use in clinical practice. They were not always fit for purpose. For example, the Nijmegen questionnaire was developed in the 1980s to assess hyperventilation; it was validated against a longer, “gold standard” instrument for that condition [91]. It subsequently became popular in respiratory clinics to diagnose or exclude breathing pattern disorder (a condition in which the normal physiological pattern of breathing becomes replaced with less efficient, shallower breathing [92]), so much so that the researchers who developed the instrument published a paper to warn fellow researchers that it had *not* been validated for this purpose [93]. Whilst a validated 17-item instrument for breathing pattern disorder (the Self-Evaluation of Breathing Questionnaire [94]) does exist, it is not in widespread clinical use. Most clinics in LOCOMOTION used Nijmegen either on all patients (e.g. as part of a comprehensive initial assessment, especially if the service had begun as a respiratory follow-up clinic) or when breathing pattern disorder was suspected.

In sum, the use of outcome measures in long covid clinics was a compromise between standardization and contingency. On the one hand, all clinics accepted the need to use “validated” instruments consistently. On the other hand, there were sometimes good reasons why they deviated from agreed practice, including mismatch between the clinic’s priorities as a research site, its priorities as a clinical service, and the particular clinical needs of a patient; the clinic’s—and the clinician’s—specialist focus; and long-held traditions of using particular instruments with which staff and patients were familiar.

### Example quality topic 2: Postural orthostatic tachycardia syndrome (POTS)

Palpitations (common in long covid) and postural orthostatic tachycardia syndrome (POTS, a disproportionate acceleration in heart rate on standing, the assumed cause of palpitations in many long covid patients) was the top priority for quality improvement identified by our patient advisory group. Reflecting discussions and evidence (of various kinds) shared in online patient communities, the group were confident that POTS is common in long covid patients and that many cases remain undetected (perhaps misdiagnosed as anxiety). Their request that all long covid patients should be “screened” for POTS prompted a search for, and synthesis of, evidence (which we published in the BMJ [95]). In sum, that evidence was sparse and contested, but, combined with standard practice in specialist clinics, broadly supported the judicious use of the NASA Lean Test [96]. This test involves repeated measurements of pulse and blood pressure with the patient first lying and then standing (with shoulders resting against a wall).

The patient advisory group’s request that the NASA Lean Test should be conducted on all patients met with mixed responses from the clinics. In site F, the lead physician had an interest in autonomic dysfunction in chronic fatigue and was keen; he had already published a paper on how to adapt the NASA Lean Test for self-assessment at home [97]. Several other sites were initially opposed. Staff at site E, for example, offered various arguments:

- The test is time-consuming, labor-intensive, and takes up space in the clinic which has an opportunity cost in terms of other potential uses;
- The test is unvalidated and potentially misleading (there is a high incidence of both false negative and false positive results);
- There is no proven treatment for POTS, so there is no point in testing for it;
- It is a specialist test for a specialist condition, so it should be done in a specialist clinic where its benefits and limitations are better understood;
- Objective testing does not change clinical management since what we treat is the patient’s symptoms (e.g. by a pragmatic trial of lifestyle measures and medication);
- People with symptoms suggestive of dysautonomia have already been “triaged out” of this clinic (that is, identified in the initial telephone consultation and referred directly to neurology or cardiology);
- POTS is a manifestation of the systemic nature of long covid; it does not need specific treatment but will improve spontaneously as the patient goes through standard interventions such as active pacing, respiratory physical therapy and sleep hygiene;
- Testing everyone, even when asymptomatic, runs counter to the ethos of rehabilitation, which is to “de-medicalize” patients so as to better orient them to their recovery journey.

When clinics were invited to implement the NASA Lean Test on a consecutive sample of patients to resolve a dispute about the incidence of POTS (from “we’ve only seen a handful of people with it since the clinic began” to “POTS is common and often missed”), all but one site agreed to participate. The tertiary POTS centre linked to site H was already running the NASA Lean Test as standard on all patients. Site C, which operated entirely virtually, passed the work to the referring general practitioner by making this test a precondition for seeing the patient; site D, which was largely virtual, sent instructions for patients to self-administer the test at home.

The NASA Lean Test study has been published separately [98]. In sum, of 277 consecutive patients tested across the eight clinics, 20 (7%) had a positive NASA Lean Test for POTS and a further 28 (10%) a borderline result. Six of 20 patients who met the criteria for POTS on testing had no prior history of orthostatic intolerance. The question of whether this test should be used to “screen” all patients was not answered definitively. But the experience of participating in the study persuaded some sceptics that postural changes in heart rate could be severe in some long covid patients, did not appear to be fully explained by their previously held theories (e.g. “functional”, anxiety, deconditioning), and had likely been missed in some patients. The outcome of this particular quality improvement cycle was thus not a wholescale change in practice (for which the evidence base was weak) but a more subtle increase in clinical awareness, a greater willingness to *consider* testing for POTS and a greater commitment to contribute to research into this contested condition.

More generally, the POTS audit prompted some clinicians to recognize the value of quality improvement in novel clinical areas. One physician who had initially commented that POTS was not seen in their clinic, for example, reflected:

“*Our clinic population is changing. […] Overall there’s far fewer post-ICU patients with ECMO [extra-corporeal membrane oxygenation] issues and far more long covid from the community, and this is the bit our clinic isn’t doing so well on. We’re doing great on breathing pattern disorder; neuro[logists] are helping us with the brain fogs; our fatigue and occupational advice is ok but some of the dysautonomia symptoms that are more prevalent in the people who were not hospitalized – that’s where we need to improve*.”

-Respiratory physician, site G (from field visit 6.6.23)

### Example quality topic 3: Management of fatigue

Fatigue was the commonest symptom overall and a high priority among both patients and clinicians for quality improvement. It often coexisted with the cluster of neurocognitive symptoms known as brain fog, with both conditions relapsing and remitting in step. Clinicians were keen to systematize fatigue management using a familiar clinical framework oriented around documenting a full clinical history, identifying associated symptoms, excluding or exploring comorbidities and alternative explanations (e.g. poor sleep patterns, depression, menopause, deconditioning), assessing how fatigue affects physical and mental function, implementing a program of physical and cognitive therapy that was sensitive to the patient’s condition and confidence level, and monitoring progress using validated patient-reported outcome measures and symptom diaries.

The underpinning logic of this approach, which broadly reflected World Health Organization guidance [99], was that fatigue and linked cognitive impairment could be a manifestation of many—perhaps interacting—conditions but that a whole-patient (body and mind) rehabilitation program was the cornerstone of management in most cases. Discussion in the quality improvement collaborative focused on issues such as whether fatigue was so severe that it produced safety concerns (e.g. in a person’s job or with childcare), the pros and cons of particular online courses such as yoga, relaxation and mindfulness (many were viewed positively, though the evidence base was considered weak), and the extent to which respiratory physical therapy had a crossover impact on fatigue (systematic reviews suggested that it may do, but these reviews also cautioned that primary studies were sparse, methodologically flawed, and heterogeneous [100, 101]). They also debated the strengths and limitations of different fatigue-specific outcome measures, each of which had been developed and validated in a different condition, with varying emphasis on cognitive fatigue, physical fatigue, effect on daily life, and motivation. These instruments included the Modified Fatigue Impact Scale; Fatigue Severity Scale [102]; Fatigue Assessment Scale; Functional Assessment Chronic Illness Therapy—Fatigue (FACIT-F) [103]; Work and Social Adjustment Scale [104]; Chalder Fatigue Scale [105]; Visual Analogue Scale—Fatigue [106]; and the EQ5D [87]. In one clinic (site F), three of these scales were used in combination for reasons discussed below.

Some clinicians advocated melatonin or nutritional supplements (such as vitamin D or folic acid) for fatigue on the grounds that many patients found them helpful and formal placebo-controlled trials were unlikely ever to be conducted. But neurostimulants used in other fatigue-predominant conditions (e.g. brain injury, stroke), which also lacked clinical trial evidence in long covid, were viewed as inappropriate in most patients because of lack of evidence of clear benefit and hypothetical risk of harm (e.g. adverse drug reactions, polypharmacy).

Whilst the patient advisory group were broadly supportive of a whole-patient rehabilitative approach to fatigue, their primary concern was *fatiguability*, especially post-exertional symptom exacerbation (PESE, also known as “crashes”). In these, the patient becomes profoundly fatigued some hours or days after physical or mental exertion, and this state can last for days or even weeks [107]. Patients viewed PESE as a “red flag” symptom which they felt clinicians often missed and sometimes caused. They wanted the quality improvement effort to focus on ensuring that all clinicians were aware of the risks of PESE and acted accordingly. A discussion among patients and clinicians at a quality improvement collaborative meeting raised a new research hypothesis—that reducing the number of repeated episodes of PESE may improve the natural history of long covid.

These tensions around fatigue management played out differently in different clinics. In site C (the GP-led virtual clinic run from a community hub), fatigue was viewed as one manifestation of a whole-patient condition. The lead general practitioner used the metaphor of untangling a skein of wool: “you have to find the end and then gently pull it”. The underlying problem in a fatigued patient, for example, might be an undiagnosed physical condition such as anaemia, disturbed sleep, or inadequate pacing. These required (respectively) the chronic fatigue service (comprising an occupational therapist and specialist psychologist and oriented mainly to teaching the techniques of goal-setting and pacing), a “tiredness” work-up (e.g. to exclude anaemia or menopause), investigation of poor sleep (which, not uncommonly, was due to obstructive sleep apnea), and exploration of mental health issues.

In site G (a hospital clinic which had evolved from a respiratory service), patients with fatigue went through a fatigue management program led by the occupational therapist with emphasis on pacing, energy conservation, avoidance of PESE and sleep hygiene. Those without ongoing respiratory symptoms were often discharged back to their general practitioner once they had completed this; there was no consultant follow-up of unresolved fatigue.

In site F (a rehabilitation clinic which had a longstanding interest in chronic fatigue even before the pandemic), active interdisciplinary management of fatigue was commenced at or near the patient’s first visit, on the grounds that the earlier this began, the more successful it would be. In this clinic, patients were offered a more intensive package: a similar occupational therapy-led fatigue course as those in site G, plus input from a dietician to advise on regular balanced meals and caffeine avoidance and a group-based facilitated peer support program which centred on fatigue management. The dietician spoke enthusiastically about how improving diet in longstanding long covid patients often improved fatigue (e.g. because they had often lost muscle mass and tended to snack on convenience food rather than make meals from scratch), though she agreed there was no evidence base from trials to support this approach.

### Pursuing local quality improvement through MDTs

Whilst some long covid patients had “textbook” symptoms and clinical findings, many cases were unique and some were fiendishly complex. One clinician commented that, somewhat paradoxically, “easy cases” were often the post-ICU follow-ups who had resolving chest complications; they tended to do well with a course of respiratory physical therapy and a return-to-work program. Such cases were rarely brought to MDT meetings. “Difficult cases” were patients who had not been hospitalized for their acute illness but presented with a months- or years-long history of multiple symptoms with fatigue typically predominant. Each one was different, as the following example (some details of which have been fictionalized to protect anonymity) illustrates.

*The MDT is discussing Mrs Fermah, a 65-year-old homemaker who had covid-19 a year ago. She has had multiple symptoms since, including fluctuating fatigue, brain fog, breathlessness, retrosternal chest pain of burning character, dry cough, croaky voice, intermittent rashes (sometimes on eating), lips going blue, ankle swelling, orthopnoea, dizziness with the room spinning which can be triggered by stress, low back pain, aches and pains in the arms and legs and pins and needles in the fingertips, loss of taste and smell, palpitations and dizziness (unclear if postural, but clear association with nausea), headaches on waking, and dry mouth. She is somewhat overweight (body mass index 29) and admits to low mood. Functionally, she is mostly confined to the house and can no longer manage the stairs so has begun to sleep downstairs. She has stumbled once or twice but not fallen. Her social life has ceased and she rarely has the energy to see her grandchildren. Her 70-year-old husband is retired and generally supportive, though he spends most evenings at his club. Comorbidities include glaucoma which is well controlled and overseen by an ophthalmologist, mild club foot (congenital) and stage 1 breast cancer 20 years ago. Various tests, including a chest X-ray, resting and exercise oximetry and a blood panel, were normal except for borderline vitamin D level. Her breathing questionnaire score suggests she does not have breathing pattern disorder. ECG showed first-degree atrioventricular block and left axis deviation. No clinician has witnessed the blue lips. Her current treatment is online group respiratory physical therapy; a home visit is being arranged to assess her climbing stairs. She has declined a psychologist assessment. The consultant asks the nurse who assessed her: “Did you get a feel if this is a POTS-type dizziness or an ENT-type?” She sighs. “Honestly it was hard to tell, bless her.”—Site A MDT*

This patient’s debilitating symptoms and functional impairments could all be due to long covid, yet “evidence-based” guidance for how to manage her complex suffering does not exist and likely never will exist. The question of which (if any) additional blood or imaging tests to do, in what order of priority, and what interventions to offer the patient will not be definitively answered by consulting clinical trials involving hundreds of patients, since (even if these existed) the decision involves weighing *this* patient’s history and the multiple factors and uncertainties that are relevant in her case. The knowledge that will help the MDT provide quality care to Mrs Fermah is *case-based* knowledge—accumulated clinical experience and wisdom from managing and deliberating on multiple similar cases. We consider case-based knowledge further in the “Discussion”.

## Discussion

### Summary of key findings

This study has shown that a quality improvement collaborative of UK long covid clinics made some progress towards standardizing assessment and management in some topics, but some variation remained. This could be explained in part by the fact that different clinics had different histories and path dependencies, occupied a different place in the local healthcare ecosystem, served different populations, were differently staffed, and had different clinical interests. Our patient advisory group and clinicians in the quality improvement collaborative broadly prioritized the same topics for improvement but interpreted them somewhat differently. “Quality” long covid care had multiple dimensions, relating to (among other things) service set-up and accessibility, clinical provision appropriate to the patient’s need (including options for referral to other services locally), the human qualities of clinical and support staff, how knowledge was distributed across (and accessible within) the system, and the accumulated collective wisdom of local MDTs in dealing with complex cases (including multiple kinds of specialist expertise as well as relational knowledge of what was at stake for the patient). Whilst both staff and patients were keen to contribute to the quality improvement effort, the burden of measurement was evident: multiple outcome measures, used repeatedly, were resource-intensive for staff and exhausting for patients.

### Strengths and limitations of this study

To our knowledge, we are the first to report both a quality improvement collaborative and an in-depth qualitative study of clinical work in long covid. Key strengths of this work include the diverse sampling frame (with sites from three UK jurisdictions and serving widely differing geographies and demographics); the use of documents, interviews and reflexive interpretive ethnography to produce meaningful accounts of how clinics emerged and how they were currently organized; the use of philosophical concepts to analyse data on how MDTs produced quality care on a patient-by-patient basis; and the close involvement of patient co-researchers and coauthors during the research and writing up.

Limitations of the study include its exclusive UK focus (the external validity of findings to other healthcare systems is unknown); the self-selecting nature of participants in a quality improvement collaborative (our patient advisory group suggested that the MDTs observed in this study may have represented the higher end of a quality spectrum, hence would be more likely than other MDTs to adhere to guidelines); and the particular perspective brought by the researchers (two GPs, a physical therapist and one non-clinical person) in ethnographic observations. Hospital specialists or organizational scholars, for example, may have noticed different things or framed what they observed differently.

### Explaining variation in long covid care

Sutherland and Levesque’s framework mentioned in the “Background” section does not explain much of the variation found in our study [70]. In terms of capacity, at the time of this study most participating clinics benefited from ring-fenced resources. In terms of evidence, guidelines existed and were not greatly contested, but as illustrated by the case of Mrs Fermah above, many patients were exceptions to the guideline because of complex symptomatology and relevant comorbidities. In terms of agency, clinicians in most clinics were passionately engaged with long covid (they were pioneers who had set up their local clinic and successfully bid for national ring-fenced resources) and were generally keen to support patient choice (though not if the patient requested tests which were unavailable or deemed not indicated).

Astma et al.’s list of factors that may explain variation in practice (see “Background”) includes several that may be relevant to long covid, especially that the definition of appropriate care in this condition remains somewhat contested. But lack of opportunity to discuss cases was not a problem in the clinics in our sample. On the contrary, MDT meetings in each locality gave clinicians *multiple* opportunities to discuss cases with colleagues and reflect collectively on whether and how to apply particular guidelines.

The key problem was not that clinicians disputed the guidelines for managing long covid or were unaware of them; it was that *the guidelines were not self-interpreting*. Rather, MDTs had to *deliberate* on the balance of benefits and harms in different aspects of individual cases. In patients whose symptoms suggested a possible diagnosis of POTS (or who suspected themselves of having POTS), for example, these deliberations were sometimes lengthy and nuanced. Should a test result that is not technically in the abnormal range but close to it be treated as diagnostic, given that symptoms point to this diagnosis? If not, should the patient be told that the test *excludes* POTS or that it is equivocal? If a cardiology opinion has stated firmly that the patient does not have POTS but the cardiologist is not known for their interest in this condition, should a second specialist opinion be sought? If the gold standard “tilt test” [108] for POTS (usually available only in tertiary centres) is not available locally, does *this* patient merit a costly out-of-locality referral? Should the patient’s request for a trial of off-label medication, reflecting discussions in an online support group, be honoured? These are the kinds of questions on which MDTs deliberated at length.

The fact that many cases required extensive deliberation does not necessarily justify variation in practice among clinics. But taking into account the clinics’ very different histories, set-up, and local referral pathways, the variation begins to make sense. A patient who is being assessed in a clinic that functions as a specialist chronic fatigue centre and attracts referrals which reflect this interest (e.g. site F in our sample) will receive different management advice from one that functions as a telephone-only generalist assessment centre and refers on to other specialties (site C in our sample). The wide variation in case mix, coupled with the fact that a different proportion of these cases were highly complex in each clinic (and in different ways), suggests that variation in practice may reflect appropriate rather than inappropriate care.

Our patient advisory group affirmed that many of the findings reported here resonated with their own experience, but they raised several concerns. These included questions about patient groups who may have been missed in our sample because they were rarely discussed in MDTs. The decision to take a case to MDT discussion is taken largely by a clinician, and there was evidence from online support groups that some patients’ requests for their case to be taken to an MDT had been declined (though not, to our knowledge, in the clinics participating in the LOCOMOTION study).

We began this study by asking “what is quality in long covid care?”. We initially assumed that this question referred to a generalizable evidence base, which we felt we could identify, and we believed that we could then determine whether long covid clinics were following the evidence base through conventional audits of structure, process, and outcome. In retrospect, these assumptions were somewhat naïve. On the basis of our findings, we suggest that a better (and more individualized) research question might be “to what extent does each patient with long covid receive evidence-based care appropriate to their needs?”. This question would require individual case review on a sample of cases, tracking each patient longitudinally including cross-referrals, and also interviewing the patient.

### Nomothetic versus idiographic knowledge

In a series of lectures first delivered in the 1950s and recently republished [109], psychiatrist Dr Maurice O’Connor Drury drew on the later philosophy of his friend and mentor Ludwig Wittgenstein to challenge what he felt was a concerning trend: that the nomothetic (generalizable, abstract) knowledge from randomized controlled trials (RCTs) was coming to over-ride the idiographic (personal, situated) knowledge about particular patients. Based on Wittgenstein’s writings on the importance of the particular, Drury predicted—presciently—that if implemented uncritically, RCTs would result in worse, not better, care for patients, since it would go hand-in-hand with a downgrading of experience, intuition, subjective judgement, personal reflection, and collective deliberation.

Much conventional quality improvement methodology is built on an assumption that nomothetic knowledge (for example, findings from RCTs and systematic reviews) is a higher form of knowing than idiographic knowledge. But idiographic, case-based reasoning—despite its position at the very bottom of evidence-based medicine’s hierarchy of evidence [110]—is a legitimate and important element of medical practice. Bioethicist Kathryn Montgomery, drawing on Aristotle’s notion of *praxis*, considers clinical practice to be an example of case-based reasoning [111]. Medicine is governed not by hard and fast laws but by competing maxims or *rules of thumb*; the essence of judgement is deciding which (if any) rule should be applied in a particular circumstance. Clinical judgement incorporates science (especially the results of well-conducted research) and makes use of available tools and technologies (including guidelines and decision-support algorithms that incorporate research findings). But rather than being determined solely by these elements, clinical judgement is guided both by the scientific evidence *and* by the practical and ethical question “what is it best to do, for *this* individual, given *these* circumstances?”.

In this study, we observed clinical management of, and MDT deliberations on, hundreds of clinical cases. In the more straightforward ones (for example, recovering pneumonitis), guideline-driven care was not difficult to implement and such cases were rarely brought to the MDT. But cases like Mrs Fermah (see last section of “Results”) required much discussion on *which* aspects of *which* guideline were in the patient’s best interests to bring into play at any particular stage in their illness journey.

## Conclusions

One systematic review on quality improvement collaboratives concluded that “*[those] reporting success generally addressed relatively straightforward aspects of care, had a strong evidence base and noted a clear evidence-practice gap in an accepted clinical pathway or guideline”* (page 226) [60]. The findings from this study suggest that to the extent that such collaboratives address clinical cases that are *not* straightforward, conventional quality improvement methods may be less useful and even counterproductive.

The question “what is quality in long covid care?” is partly a philosophical one. Our findings support an approach that recognizes and values *idiographic knowledge*—including establishing and protecting a safe and supportive space for deliberation on individual cases to occur and to value and draw upon the collective learning that occurs in these spaces. It is through such deliberation that evidence-based guidelines can be appropriately interpreted and applied to the unique needs and circumstances of individual patients. We suggest that Drury’s warning about the limitations of nomothetic knowledge should prompt a reassessment of policies that rely too heavily on such knowledge, resulting in one-size-fits-all protocols. We also cautiously hypothesize that the need to centre the quality improvement effort on idiographic rather than nomothetic knowledge is unlikely to be unique to long covid. Indeed, such an approach may be particularly important in any condition that is complex, unpredictable, variable in presentation and clinical course, and associated with comorbidities.

## Data Availability

Selected qualitative data (ensuring no identifiable information) will be made available to formal research teams on reasonable request to Professor Greenhalgh at the University of Oxford, on condition that they have research ethics approval and relevant expertise. The quantitative data on NASA Lean Test have been published in full in a separate paper [98].

## Abbreviations

CFS: Chronic fatigue syndrome
CL: Cassie Lee
EL: Emma Ladds
ICU: Intensive care unit
JCS: Jenny Ceolta-Smith
JLD: Julie Darbyshire
LOCOMOTION: LOng COvid Multidisciplinary consortium Optimising Treatments and services across the NHS
MDT: Multidisciplinary team
ME: Myalgic encephalomyelitis
MERS: Middle East Respiratory Syndrome
NASA: National Aeronautics and Space Association
OT: Occupational therapy/ist
PESE: Post-exertional symptom exacerbation
POTS: Postural orthostatic tachycardia syndrome
SALT: Speech and language therapy
SARS: Severe Acute Respiratory Syndrome
TG: Trisha Greenhalgh
UK: United Kingdom
US: United States
WHO: World Health Organization
